# Residual Stress Measurement Techniques for Metal Joints, Metallic Coatings and Components in the Railway Industry: A Review

**DOI:** 10.3390/ma16010232

**Published:** 2022-12-27

**Authors:** Olivia Kendall, Anna Paradowska, Ralph Abrahams, Mark Reid, Cong Qiu, Peter Mutton, Wenyi Yan

**Affiliations:** 1 Department of Mechanical and Aerospace Engineering, Monash University, Clayton, VIC 3800, Australia; 2 Australian Nuclear Science and Technology Organisation, Lucas Heights, NSW 2234, Australia; 3 School of Civil Engineering, The University of Sydney, Sydney, NSW 2006, Australia; 4 Institute of Railway Technology, Monash University, Clayton, VIC 3800, Australia

**Keywords:** residual stress, measurement, railway, steel, destructive, non-destructive, welding, laser cladding

## Abstract

Manufacturing and maintenance procedures in the railway industry regularly implement welding and metal deposition operations to produce joints, coatings and repair structures. During these processes, residual stresses arise through the generation of heat affected zones and plastic deformation. This makes accurate measurements of the internal stresses a critical aspect of manufacturing, monitoring, repair and model validation in the develop new metallic coating and joining technologies. Selection of an appropriate residual stress measurement method has many important factors including component size, resolution and the magnitude and location of internal stresses, often resulting in a combination of techniques required to obtain complete assessment of the stress state. This paper offers a review of residual stress measurement techniques for railway components including rail joints and coatings through comparison of destructive and non-destructive approaches, their measurement capabilities, benefits and limitations. A comprehensive discussion of different applications is provided with a summary of facilities available to both research and industry.

## 1. Introduction

Railway transport is a rapidly growing, 500-billion-dollar global industry and one of the largest contributors to the world economy. Most countries rely on a combination of light, metro, freight and high-speed railway for the transportation of minerals, goods and people. These networks can be thousands of kilometres in length and incorporate underground, above ground and elevated railway systems that traverse seas, mountains and deserts. Over 4 million metric tonnes of steel are exported annually by the railway industry for use in tracks, rollingstock and large structural components. The selection of steel grades for these parts depends heavily on the required mechanical properties for the application. Hypereutectoid steel grades are used by the heavy haul industry as the high carbon content increases the rail strength to withstand the high tonnage conditions [1]. In comparison, steels with lower carbon levels are preferred for urban, light rail networks which require sharp rail curvatures and have lower loading requirements for passenger transit. Specialised components such as railway crossings call for wear resistant materials to prolong the operational life and often use manganese steels, whilst axles can be manufactured from high strength low alloy steels for high-speed railway applications [2]. Railway wheels are commonly manufactured from similar carbon steels to provide wear, rolling contact fatigue and thermal loading resistance during operation. Aluminium is also increasingly used in the body structures of rollingstock as a cost-effective means to reduce weight whilst meeting strength and safety requirements. In addition, these networks are accompanied by extensive infrastructure including railway bridges, gantries, cantilever systems and overhead wiring structures. These components withstand high structural and mechanical loads to support signalling equipment and catenary systems that ensure continous railway operation. Pantograph/catenary systems provide an energy supply throughout the network facilitating the operation of electric trains. These structures face their own significant environmental challenges such as wind loading which can cause large wire deflections that may affect operation and lead to fatigue failure [3]. The wear of the contact materials due to rapid temperature changes and electrical discharge is also of ongoing concern in these components [4].

**Figure 1:**
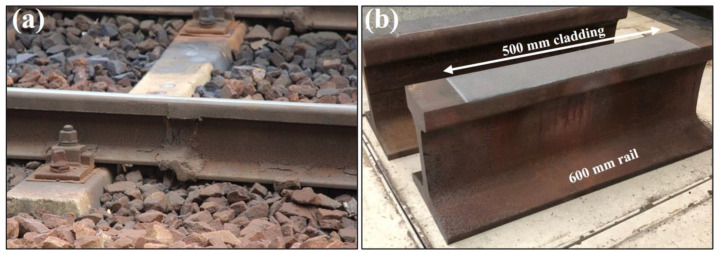
(**a**) Thermite welded rail by Bombarda et al. [5], (**b**) laser cladding deposition on rail by Kendall et al. [6].

The production, maintenance, repair and replacement of this infrastructure relies on metal joining and coating technologies, as shown in Figure 1, to integrate new components into networks and to recondition existing parts. Moreover, the safe operation of railway systems depends upon the production of high-quality joints and deposition of coatings with known properties that meet industry standards. 

**Figure 2:**
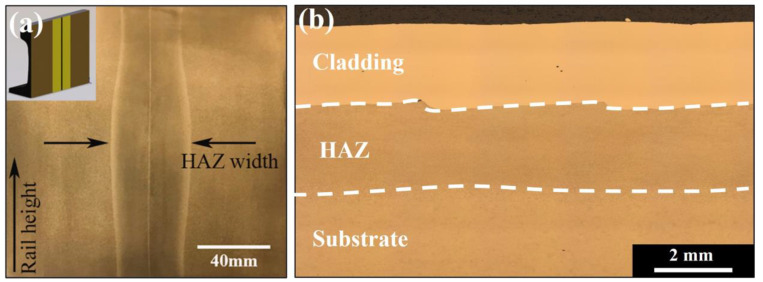
Generation of HAZ after (**a**) Flash-butt welding by Micheletto et al. [17] and (**b**) laser metal deposition.

Welding is a widely implemented technique for in situ joining and surface repair during railway production using aluminothermic, flash-butt and arc welding [7,8,9,10,11,12]. This technology is employed in the assembly of bogie frames, joining of rail track through continuous welding and in the replacement of individual sections of damaged rail. Surface reconditioning is performed using overlay welding whilst aluminium friction stir welds can be found in rollingstock components. As this technique can be easily implemented and is a reliable method of joining critical infrastructure such as pipelines, pressure vessels and structural steel components, welding continues to be used across many industries. The welding process requires high heat inputs that form large thermal gradients which cause localised melting, solidification shrinkage, distortion and phase transformations in the heat affected zone (HAZ) [13,14,15]. These changes influence the mechanical properties and introduce residual stresses. High tensile residual stresses are undesirable as it increases the likelihood of fatigue cracking and failure. Along with thermal inputs, the selection of deposition and filler materials also contributes to the generation and redistribution of internal stresses [16]. A flash butt-welded rail join is shown in Figure 2a. This technique is commonly used to produce continuous rail track. As the two molten rail ends are pushed together, a heat affected region adjacent to this weld centreline is produced. Heat dissipates away from the fusion boundary into the bulk rail causing solid-state microstructural changes. This region extends into the two neighbouring rail ends and produces the wide HAZ seen below in Figure 2a. It has been reported that this heat modified regions can be greater than 45 mm wide in welded rail thereby introducing a large area of variable residual stress and microstructure [10]. Therefore, this method must be carefully controlled and well understood to avoid the introduction and propagation of defects.

More recently, additive-manufacturing based technologies such as laser metal deposition (LMD) and laser cladding have been applied to remanufacture components through depositing a hard facing layer. This has been implemented to produce high wearing coatings for the mining industry and is now studied as a maintenance strategy for railway components [1,18]. These processes use a high energy laser to melt a metallic powder or wire at the substrate surface which cools to form a metallurgically bonded deposition and is compatible with a range of metals and alloys including steel, Stellite, Hastelloy, Inconel and aluminium [19,20,21,22]. Mortazavian et al. [23] investigated laser powder deposition in railway and applied six layers of 304L to achieve a complete reconstruction of a rail profile.

In comparison to welding techniques, LMD and laser cladding require lower thermal inputs that reduce the size of the heat affected regions. These have been reported to be around 2 mm wide shown in Figure 2b, in comparison to the large regions produced during welding techniques. Due to consecutive melting and solidification cycles, residual stresses arise in the coating and the adjacent HAZ. The use of dissimilar coating alloys may further contribute to a complex residual stress state from differences in the chemical composition, thermal expansion coefficient and mismatch of mechanical properties [24]. Laser cladding is regularly used to apply coatings to high wearing components in the mining industry and has been investigated as a coating technique to increase the wear resistance and fatigue life of rail. Additionally, LMD is widely studied as a method for localised repairs for railheads, axles and wheels whilst thermal spray techniques are used to apply molybdenum coatings to railway axles [25].

Understanding the causes of stress generation during a thermal process is critical to avoid an undesirable stress state but also may be used to purposefully engineer stresses into a component to improve performance. This is achieved by controlling the materials, cooling conditions or applying thermal treatment after metal deposition. Unal et al. [26] used shot peening on a medium carbon railway axle to introduce compressive surface stresses through deformation. It was found severe shot peening was the most effective at increasing the high cycle fatigue lifetime whilst re-shot peening had greater effect at prolonging low cycle fatigue. Li et al. [27] applied laser shock peening to a flash-butt welded high speed rail and successfully improved the fatigue properties. This was attributed to the introduction of compressive stresses after laser shock peening due to an increase in dislocation density.

Accurately measuring the stresses generated after joining and coating processes is essential as high tensile residual stresses increase the likelihood of failure, deformation, coating delamination and a shortened operational life. A high internal stress state increases the susceptibility of defect formation and promotes unpredictable behaviour in high-wearing, high pressure and load-bearing infrastructure [28,29]. This was demonstrated by Nassiraei et al. [30,31] who investigated stress concentration factors (SCFs) in welded X-joints as the presence of stress concentrations near welds negatively effects the fatigue lifetime. FE models created to determine SCFs in these complex welded structures not only play a critical role in predicting performance but also established how stresses could be successfully reduced in existing welds with the addition of reinforced laminates at the weld site. During railway operation it is the combination of residual stress and wheel-rail contact stress which determines the fatigue behaviour. Due to the unique characteristics of metallic materials such as the crystallographic lattice structure and magnetic properties, there are many standard techniques widely available and applied in research and industry to assess residual stresses in metal components. This selection can depend on a number of factors including, internal or surface measurements, coating thickness sand sample size.

This paper presents the main techniques currently available to research and industry to determine residual stresses in railway components. An overview of the applications of residual stress measurement during the manufacturing, welding and operation of rail components including track, wheels, axles, crossings and bogies is presented. This is followed by a description of destructive and non-destructive approaches with a summary of some of the available measurement services and facilities. The physical characteristics and applications of each measurement method are provided to establish the most appropriate and accurate technique depending on the significance of stress and component size. A comparison of the accuracy and variation, advantages, disadvantages, available codes and standards are highlighted for the most common techniques used to determine residual stress in the railway industry. A final conclusion is given on the selection of residual stress measurement techniques which is highly dependent on the accuracy required, cost and availability. These techniques can be used in combination to address the limitations of individual methods and provide a comprehensive analysis of residual stresses in larger railway components.

## 2. Residual Stress Measurement Techniques for Railway Components

Experimentally obtained residual stress data is essential to assess mechanical performance, validate numerical models, develop new technologies and to monitor and maintain infrastructure. A significant application of railway residual stress measurements is in the manufacture of railway components [32]. Residual stresses are assessed after manufacture to ensure components meet industry requirements and establish the effect of forming and thermal processes on the internal strain. Rail wheels are required to contain compressive stresses in the wheel rim to restrict crack propagation whilst rails are known to contain tensile stress in the rail head that is met with compression under wheel contact [33]. Thermal processes such as welding, and laser remanufacturing have a significant effect on stress state due to heat inputs. Residual stress measurements also play a critical role in fatigue life assessment as the internal stress state evolves during in-service loading. Experimentally obtained residual stress measurements are regularly incorporated in finite element analysis (FEA) to improve simulations that optimise processes or are used for model validation to better our understanding of rail contact mechanics.

Residual stress occurs over three length scales therefore measurement techniques must have appropriate resolution for the application. Long range stresses arise from macrostrains and are classified as Type I. Type II refers to stresses that self-equilibrate over a length scale equivalent to the size of an individual grain in the microstructure. Stresses that occur within a single grain at an atomic scale are Type III stresses which may arise from dislocations or crystal lattice inhomogeneities [34]. The nature and origins of residual stress is described in detail in [35].

**Figure 3:**
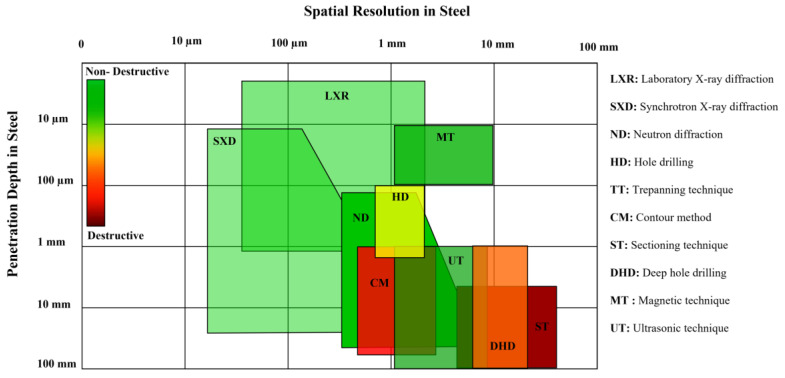
Spatial resolution and penetration depth of destructive and non-destructive residual stress measurement techniques for steel.

Destructive and semi-destructive techniques include strain gauge, hole drilling and the contour method which rely on machining to release elastic strain in the lattice used to calculate the corresponding stress [36]. These approaches measure displacement of the cut surface or apply strain gauges to determine changes in strain which is then used to indirectly calculate stress using Hooke’s law. These methods are more accessible and easily implemented, however have limitations associated with the destruction caused by the inherent requirement for material removal, lower resolution and reduced measurement capability at increasing subsurface depths. This is shown in Figure 3 which presents a comparison of spatial resolution and measurement depth of destructive and non-destructive techniques for steel. Non-destructive techniques include diffraction-based methods that use neutron, X-ray and synchrotron sources as well as ultrasonic and magnetic methods. These correlate strain with the measurement of another material parameter such as lattice spacing, magnetism or movement of ultrasonic waves through the material [37]. For example, internal stresses result in a shift in the diffraction pattern when compared to a strain free reference. The calculated strain caused by changes in the lattice parameter at the measurement location is then used to calculate the principal stresses. In comparison, the presence of internal stress effects how a magnetic field or ultrasonic wave passes through a material. The changes in output signal compared to a reference sample or calibration curves is then used to determine the internal stress state. Non-destructive approaches have the advantage of high accuracy and resolution and ability to perform surface and internal measurements. This is at the expense of longer data collection and processing times as well as the requirement for specialised instruments and facilities that are less accessible. A high resolution is desirable for residual stress measurements and a high spatial resolution can be achieved when the measurement depth is small, as indicated in Figure 3. With increasing measurement depth, the attainable resolution generally decreases, even using non-destructive approaches, as the objects become larger. Therefore, it is important to choose the optimal approach for the given component with consideration of the cost, measurement time, accessibility and intended use of the output data. The method of measurement may also depend on size of component, accessibility to measurement location, required resolution and level of expertise.

### 2.1. Destructive Methods

Destructive techniques are a well-established approach for stress evaluation and have the advantage of being highly accessible, accurate and capable of both surface and internal measurements. The mechanical method can be classified as either destructive or semi-destructive depending upon the extent of machining at the measurement site. This approach relies on evaluating the residual stresses through determining the change in strain or deformation brought about by stress relaxation after material removal. Strain relaxation is then measured with a strain gauge or using laser optics for higher resolution readings.

#### 2.1.1. Sectioning

One of the earliest measurement techniques developed for residual stress analysis is Sectioning method. This approach applies electronic or mechanical strain gauges to the component surface before destructively cutting cross sections from the larger specimen. The deformation caused by a release of stress registers as a change in strain, which is measured during cutting and used to calculate the residual stress in that direction.

**Figure 4:**
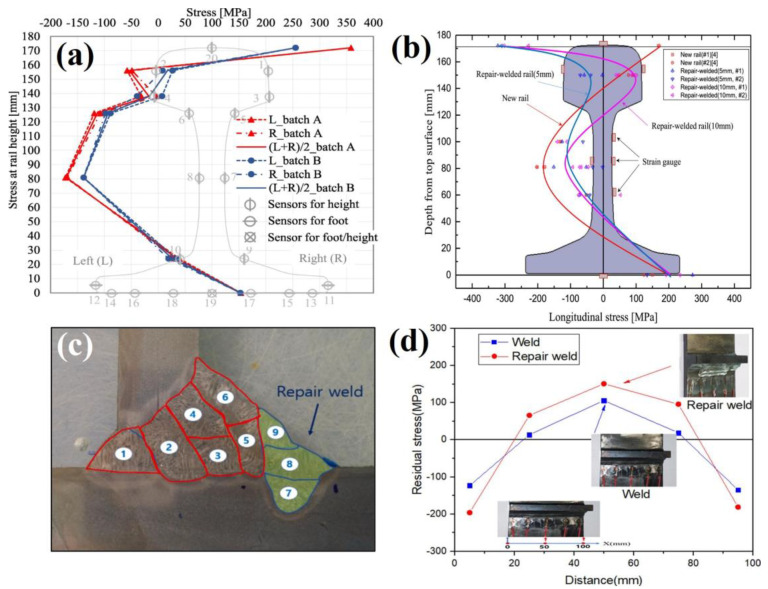
(**a**) Residual stress profile after sectioning a rail head Kang et al. [38] (**b**) Stress profile comparison of new and repair welded rails by Jun et al. [39]. A study by Seo et al. [42] showing a (**c**) weld repair on a bogie frame and (**d**) stress comparison of a repaired and unrepaired weld.

The sectioning technique was employed by Kang et al. [38] to determine the internal stress state after manufacturing UIC 60 rails. The measurements were carried out according to the EN 13674-1 standard using 1100 mm length sections. The longitudinal and transverse stresses were measured by positioning 20 strain gauges across a 20 mm cross section as shown in Figure 4a. A characteristic ‘C’ shaped stress profile was obtained across the rail cross section, transitioning from tension at the surface to compression in the web before returning to tension at the foot. The transverse stress across the foot of the rail was also found to vary from 156 MPa in tension to 56 MPa compression between the centre and edge. This technique was supplemented by X-ray measurements, however due to the effects of surface roughness, the stresses obtained using sectioning showed a better agreement with literature. For this reason, the sectioning stresses were used to establish a relationship with the bending fatigue resistance. As shown in Figure 4a the number of measurement points depends upon the number of strain gauges applied and the results may be influenced by plastic deformation during cutting or thermal inputs. A comprehensive study by Jun et al. [39] measured the stress in rails repaired by arc welding using a similar sectioning approach. In comparison to the as-manufactured stresses, welding was found to induce compressive stresses in the rail head after repairs with 5 mm and 10 mm weld depths. This was determined by positioning strain gauges at eight locations at either side of the rail head, web and foot, from a section extracted from the centre of the weld site, before releasing the stress in the longitudinal, vertical and axial directions. These findings were used perform FEA that takes into consideration the solid-state phase transformation induced by welding.

The sectioning method has also proven to be a very effective approach for stress determination in large components such as railway axles. Rieger et al. [40] used a strain gauge technique to measure residual stresses in wheel-set axles to ascertain the stress behaviour during crack propagation. Application of crack tip strain gauges applied to a notched sample were able to provide an approximation of stress near the crack tip which was used to understand crack growth and fatigue behaviour. A similar crack growth investigation on rail axles was also undertaken by Schindler et al. [41]. Seo et al. [42] used this cutting method to investigate the integrity of repair welds in a gas metal arc welded bogie frame shown Figure 4c. Strain gauges were attached at five locations near the 60 mm long weld toe repair site. The weld repair exhibited higher compressive and tensile stresses at all measurement sites compared to an unrepaired weld as shown in Figure 4d. These data were used to validate a FEA model using heat transfer and thermal stress analysis with consideration of the distributed heat flux and latent heat to capture the residual stresses in complex welded components. This was used to understand the influence in welding pass direction and weld repair width on the generation of internal stresses. Whilst the sectioning technique is readily implemented to inspect weld sites on larger components, this method is less suited for capturing small scale stress changes from thermal coating processes. For this reason, sectioning can be considered a lower resolution technique so is often paired with a complimentary stress measurement method.

#### 2.1.2. Hole Drilling

Hole drilling is one of the most widely implemented methods for determining residual stresses as it is a practical technique that can be performed in situ with a relatively high accuracy. This approach traditionally applies a strain gauge rosette to the workpiece to measure the equilibrium stress state before drilling a central blind hole, generally 1–5 mm in diameter [43]. The relaxation and redistribution of stress is registers as changes in strain and these values are applied to elastic theory equations with the appropriate calibration constants to determine the average magnitude of the biaxial residual stress. As it is a surface measurement method, hole drilling is considered to be semi-destructive and is regularly used to determine stresses in coatings applied through direct metal deposition and spray techniques.

**Figure 5:**
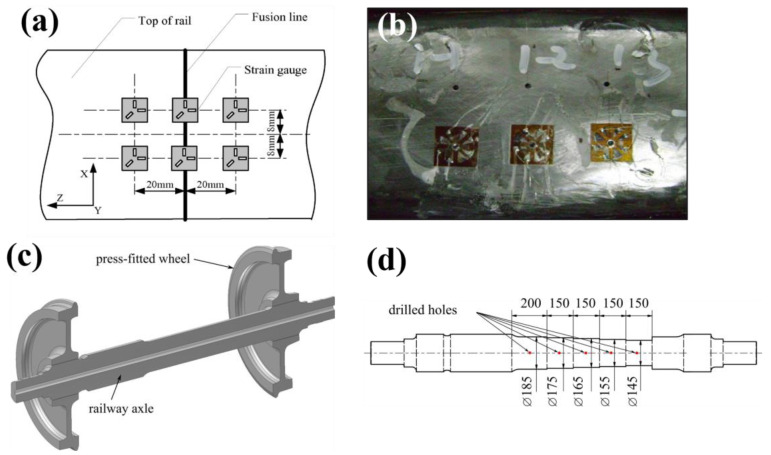
A study by Ma et al. [44] showing (**a**) Hole drilling measurement locations around a weld and (**b**) Hole drilling strain gauges applied to the rail head. A study by Pokorny et al. [47] showing a (**c**) schematic of railway axle and wheel configuration and (**d**) measurement locations after turning to reduce axle diameter for internal measurements.

Ma et al. [44] applied hole drilling to measure the residual stress across two 1000 mm sections of U71Mn rail joined by flash-butt welding. Comprehensive measurements were taken at the fusion line and 20 mm away using six measurement locations on the rail head, three along the web and another six at the rail foot shown in Figure 5a,b. These stress measurements were used in a 3D FE model of a quarter of the weld join in a 1000 mm rail incorporating an iterative substitute method and a material model describing solid state phase transformations. This was used to identify the critical role of phase transformations in stress generation during flash-butt welding and fatigue life assessment.

Whilst this technique was effective for surface stress measurements, it was noted that the steep stress gradient at the fusion boundary determined by FE analysis could not be captured using the hole-drilling technique due to limitations of the resolution. Zhu et al. [45] implemented hole drilling techniques to investigate the influence of welding on aluminium train cars used in high-speed rail. After welding two 8 mm thick aluminium plates, hole drilling was performed at 6 locations up to 30 mm from the weld site. This showed peak stresses of 130 MPa occurring at and adjacent to the weld site and which was used to validate.

FE simulations using a double ellipsoide heat source model of welded high-speed rail components. Hole drilling was also applied to maglev welded rails by Rao et al. [46] to determine the effectiveness of vibration technology for stress relief. Semi-destructive hole drilling measurements were taken at six locations along the weld site on a 3 m rail with a total drilling depth of 2 mm using a 0.2 mm/min drilling rate. Repeating stress measurements before and after vibratory stress relief on the maglev component indicated a 30% reduction in welding stress was achieved, suggesting vibration as a suitable method for stress relief when thermal processes are unsuitable.

Pokorny et al. [47] implemented a hole drilling approach to assess the fatigue life of heat-treated and induction hardened railway axles shown in Figure 5c. Axial and hoop surface stresses were recorded using a 3-grid strain gauge rosette however were limited to a depth of 2.5 mm below the axle surface. To achieve deeper measurements, sections of the axle were incrementally turned to remove 5 mm from the surface and strain was remeasured using hole drilling to obtain stresses at increasing depths as shown in Figure 5d. These measurements required post processing using FE analysis to compensate for the loss of residual stress due to the machining operations and were used to demonstrate the benefit of induction hardening in high wearing railway components.

Another approach to obtain internal stress measurements is through incremental hole drilling which takes into consideration the non-uniform distribution of stress below the surface by facilitating through thickness measurements. Strain is recorded at intermittent intervals during machining to obtain the stress at increasing depths. Whilst this method enables sub-surface measurements, the achievable depth it is generally restricted to be approximately equal to the diameter of the drilled hole [34,48]. For example, Narayanan et al. [49] used central hole drilling on determine residual stress generation in pearlitic rail laser clad with a martensitic steel. Strain gauges of 2 mm and 4 mm diameters were used to achieve measurement depths of 1 mm and 2 mm, with measurement increments of 0.05 mm and 0.1 mm, respectively. It was found that the 2 mm drill diameter used for incremental central hole drilling was more effective for surface measurements and showed strong agreement with results obtained using diffraction techniques. A deep hole drilling technique was also employed by drilling a reference hole and measuring the diameter before trepanning around this point. The change in diameter along the reference hole is then used to determine residual stress in the cladded sample. The larger drill diameters used in the deep hole drilling technique were reported to be unable to capture steep stress gradients in the cladding but were effective in determining the substrate stresses 4 mm below the cladding surface therefore is complementary to central hole drilling. From this study it was determined laser cladding of martensitic steel induces compressive stresses at the rail surface and the stress distribution stabilises during cyclic four point bending tests.

Stellite cladding on pearlitic rail was investigated by Ringsberg et al. [50] using hole drilling in accordance with ASTM E837-99 on twin disc samples. A depth of 100 µm at nine measurement locations was used to determine the hoop stress of the disc samples is tensile at the cladding surface. An error margin greater than 10% resulted from the high cladding hardness influencing the drilling operation, nevertheless the results were able to validate a FEM simulation of cladding and grinding processes. Whilst hole drilling techniques can be independently implemented, there are many companies which offer residual stress measurement services. Veqter Ltd. specialises in the deep hole drilling technique for residual stress measurements in engineering components. They offer a range of residual stress measurement methods including incremental central hole drilling and the ring-core method.

The Ring-Core method develops further upon the deep hole drilling technique by use of a rosette strain gauge and machining of a larger central ring around 14–60 mm in diameter. This method allows incremental measurements up to a depth of 5 mm and is reported to have a higher sensitivity compared to other hole drilling techniques as the achievable stress relaxation is greater with a smaller diameter core, however, can lead to greater destruction of the sample. Moazam et al. [51] performed a comparative study of residual stress in UIC 60 rail using the ring-core and sectioning method. Whilst both techniques identified tensile stresses in the rail foot, those obtained using the ring-core technique were 27% higher compared to those obtained using the destructive sectioning method. Despite the straightforward implementation this approach, hole drilling has reduced sensitivity at increasing depths, therefore, is most suitable for recording low level surface stresses. Further error is also introduced when the measured stress exceeds 50% of the yield stress which can facilitate local yielding and plasticity errors. The calibration coefficients can be determined both experimentally and using FE techniques as the error associated with the coefficients greatly influences the resultant stress. For higher accuracy hole drilling techniques, laser speckle interferometry or holography techniques have since been used to measure deformation with a higher degree of accuracy [52].

### 2.2. Contour Method

The contour method was first proposed by Prime [53] and is a destructive technique that uses sectioning to induce stress relaxation, producing a spatial map of the residual stress. The contour method first requires the component to be cut through a plane which relaxes the stresses normal to the surface resulting in deformation [53,54]. Displacement data across the cut surfaces can be obtained using contact methods with coordinate measurement machines (CMM) or contactless laser surface profiling depending on the geometry and sample size. The displacement measurements are input into a stress free, elastic FE model which determines the stress required to reverse the deformation which, based on Bueckner’s principle of superposition, is the residual stress [55]. This provides a 2D spatial map of the normal stress across the measured plane.

**Figure 6:**
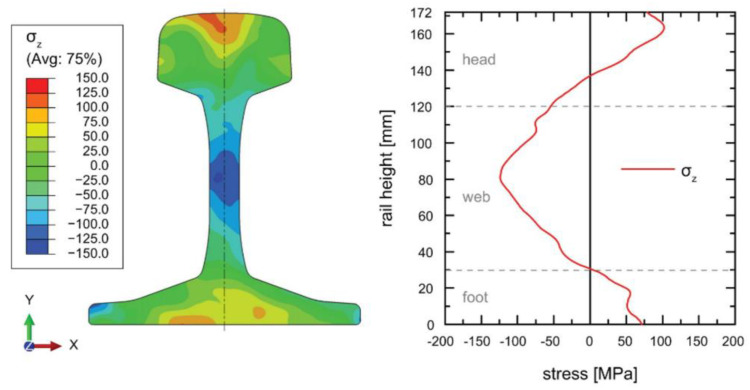
A study by Kaiser et al. [56] showing a 2D stress map and profile of a rail cross section obtained using the contour method.

Kaiser et al. [56] applied the contour technique to determine the resultant stress state in a 500 mm R260 rail after roller straightening. A 1 × 1 mm^2^ grid was used for stress calculation after measuring the deflected surfaces using CMM. The resultant stress map obtained using the strain contour measurements is presented in Figure 6. The locations of tensile stress at the rail head and foot can be readily identified with a compressive region in the web. These findings were verified using diffraction measurements and FE analysis thereby validating the simulation of the rail roller straightening procedure which can be used for process optimisation. It was noted by the authors the contour measurement process to approximately twenty hours to execute in comparison to four days of measurement required by diffraction techniques. A similar study was undertaken by Banerjee et al. [57] comparing the internal stress state of new rail with those exposed to varying degrees of loading and rail head or gauge corner wear.

The contour method has most widely been applied for stress determination in railway rails. In these components, the longitudinal direction is the largest in dimension and contains high magnitudes of stress As the contour method is a uniaxial stress measurement technique, stresses are calculated in the direction perpendicular to the cutting plane. This makes rail particularly suited to the contour technique as the longitudinal stress can readily be measured whilst providing an overview of strain across the rail head, web and foot and is also widely applied to study welded components [58,59]. The accuracy of the final stress measurements is dependent on the resolution and precision of the surface contour profiling which in turn is influenced by the cutting process and resultant surface roughness. Due to steps involved in measurement and post processing, accuracy of the results can vary greatly and several studies have reported different approaches to reduce error in contour calculations [60]. For this reason, the contour method requires expertise to accurately implement and can be accessed through professional stress testing services. For example, The StressMap group in the United Kingdom are leading specialists in the contour method and offer stress measuring services using a range of standard techniques whilst SONATS is an industrial testing firm based in France who offers contour and residual stress measurements to a worldwide customer base.

## 3. Non-Destructive Techniques

In comparison to destructive techniques such as sectioning, hole drilling and the contour method which rely on material removal to release strain, non-destructive techniques allow stress to be determined through correlation to another material parameter such as the lattice spacing, magnetic response or the ability for ultrasonic waves to pass through a material. These approaches offer higher accuracy and greater resolution without the requirement for destructive machining. The makes this non-destructive approach particularly effective for investigation of thin rail coatings, additive manufacturing techniques and in situ stress measurements for large scale rail infrastructure.

### 3.1. Diffraction Methods

Diffraction based techniques can measure internal stress accurately and non-destructively in crystalline materials such as metals, making it a very effective technique for the railway industry. Diffraction utilises the material lattice as an atomic strain gauge that correlates the elastic strain with the interplanar distance. Elastic strain produces either a contraction or expansion of the lattice spacing, resulting in a shift of the diffraction peak when compared to a strain free reference. This corresponds to a change in scattering angle which is applied to Bragg’s law to determine the principal strains that are used to calculate the residual stress indirectly using Hooke’s Law.

#### 3.1.1. Laboratory X-ray Diffraction

X-ray diffraction is a widely implemented technique for the measurement of surface stresses. This approach directs X-rays towards a polished sample which are diffracted by grains oriented to meet the Bragg scattering condition. These X-rays are collected by a rotating detector which captures the intensity at the diffracted angle. Due to the low penetration capability of X-rays in metals, this technique is limited to surface measurements of 20 µm in steel, the most common metal used by the railway industry.

**Figure 7:**
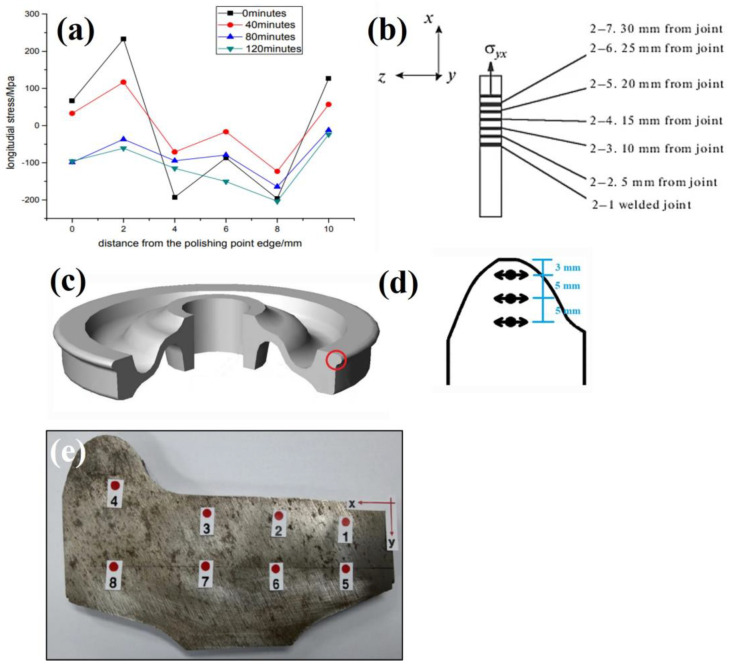
(**a**) Profiles showing the effect of electropolishing on stress by Yan et al. [61] (**b**) Strain measurement locations on a weld by Betsofen et al. [64]. A study by Rezende et al. [65] showing (**c**) a railway wheel for residual stress measurement and (**d**) measurement sites at the flange. (**e**) Stress measurement locations on an overlay welded wheel by Coo et al. [66].

Flash-butt welded U71Mn rails were investigated by Yan et al. [61] using X-ray diffraction techniques to identify stresses and determine if electropolishing can be used to reverse the stress inducing effects of grinding. Surface measurements taken at the rail head, web and foot indicated tensile transverse stresses dominate near the fusion boundary and become compressive further away from the weld site. The profiles shown in Figure 7a indicate at least 90 min of electropolishing was required to remove the grinding layer to return the surface stress state to the pre-grinding condition. Welded cruciform joints for a 16MnR train bogie were subjected to ultrasonic impact to reduce to prolong the fatigue life by Yu et al. [62]. X-ray diffraction was used to measure weld surface stresses in the longitudinal and transverse direction at three locations before and after stress relief. A reduction in stress the weld toe was achieved which initiated a transition from tensile to compressive stress in this region. This showed the influence of residual stress on the fatigue life as approximately a 92% increase in fatigue life was achieved due to microstructural changes due to ultrasonic impact at the weld site. Welding of aluminium alloys used in high speed trains was investigated by Ji et al. [63] using X-ray diffraction techniques. A heat treated 4 mm aluminium plate containing a metal inert gas weld line was used for stress measurement at seven locations over 12 mm spanning the weld, HAZ and base metal. The effect of fitting method on stress calculation was assessed, with gaussian fits generating slightly larger results than Pearson VII methods. Nevertheless, both methods showed a strong agreement with FE simulations.

Stellite cladding and welds on rail steel was studied by Betsofen et al. [64] to determine the stress distributions using X-ray diffraction. Surface measurements were taken at 12 locations at increasing distances from the weld site in the transverse and longitudinal directions shown in Figure 7b. This approach established high compressive stresses radiate from the joint which was correlated to the transformation temperature whilst cladding was shown to generate tensile stresses at the surface. The residual stresses neutralised in the cladding at 120 µm below the surface, suggesting the presence of compressive stresses deeper within the deposition.

Rezende et al. [64] used X-ray diffraction techniques to analyse the residual stress in the flange of forged railway wheels shown in Figure 7c. Different steel grades were assessed by taking a transverse cross section to measure the hoop stress at increasing distance from the flange edge shown in Figure 7d. The widely implemented sin^2^ψ method was used along with the assumption of plane stress conditions due to the limited penetration of X-rays. Using the diffraction data, phase analysis indicated the presence of bainite increased the compressive stresses in the wheel flange compared to a pearlitic-ferritic microstructure. Submerged arc welding for tread and flange restoration in rollingstock wheels was also investigated by Coo et al. [66]. X-ray diffraction was performed at the measurement locations shown in Figure 7e using a 2-dimensional detector and the cosα method which has been reported to have a reduced measurement time and is more portable equipment compared to the sin^2^ψ approach [67]. The stresses in the overlay weld shown in Figure 7e ranged from −256 to 86 MPa and were generally compressive due to the introduction of a bainitic microstructure.

The studies discussed above report the application of X-ray diffraction to railway components for surface measurements. This technique can also be employed with electropolishing to incrementally remove surface layers and achieve measurements at increasing depths. The influence of deep rolling on the susceptibility to crack propagation in railway axles was assessed by Regazzi et al. [68]. X-ray stress measurements were carried out on full scale railway axle after fatigue testing using electropolishing to measure the stresses in 100 µm increments to a depth of 2.5 mm below the surface. Compressive surface stresses introduced from deep rolling were found to prevent defect formation during crack propagation testing using an introduced 2 mm notch. Takahashi et al. [69] used a similar approach to ascertain the internal stresses in railway wheels. X-ray diffraction was performed on a 100 mm extracted section to measure the hoop stress after incrementally electropolishing to a depth of 4 mm below the surface. These findings were used to validate the elasto-plastic FE analysis used to capture the initial stress state of the wheel by determining residuals stresses due to temperature changes during manufacture. The final stress state after removal of a section via cutting was also determined and compared to experimental measurements.

#### 3.1.2. Synchrotron X-ray Diffraction

The main advantage for synchrotron energy source for X-ray diffraction is the increased penetration capability of the high energy, high intensity X-rays. This enables internal strain measurements to be performed, up to 20 mm deep in steel components. The reduced measurement time due to higher signal output allows extensive strain mapping to be carried out on railway components with a high spatial resolution capable of detecting low internal residual stress.

**Figure 8:**
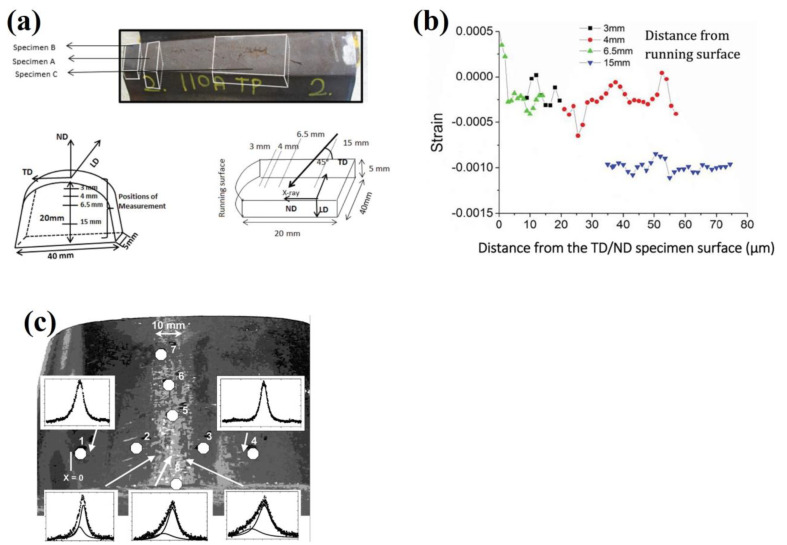
Studies by Dhar et al. [70] showing (**a**) the extraction of a sample from a railway crossing for synchrotron residuals stress analysis and (**b**) measurements taken different locations from the running surface indicating tensile and compressive strains. (**c**) Strain measurement locations on the rail head by Pyzalla et al. [73] for white etching layer analysis.

Dhar et al. [70] used synchrotron radiation to measure strains in the nose of a manganese rail crossing shown in Figure 8a. These measurements were performed on a 5 mm slice extracted from the crossing, and measurements were taken at increasing depths up to 15 mm below the rail surface. This technique verified high compressive microstrains exist at this depth due to deformation from contact loading shown in Figure 8b. This indicates the extent to which the service conditions influence the internal stress state. It was noted that for this application, synchrotron radiation was beneficial in determining surface stresses as X-ray diffraction was unable to penetrate far enough below the cut surface to a depth unaffected by cutting strain release [71]. Kelleher et al. [72] performed synchrotron X-ray strain mapping on ex-service and roller straightened railway rail. Strain scanned was performed on 5 mm cross section of pearlitic rail which effectively relieved longitudinal stress, allowing measurements to be taken in the vertical and transverse directions. This resulted in 2D stress maps for each rail slice and was used to identify critical differences in stress generation at the rail head between straightened and worn rails. A comparison with simulated results suggested increasing the sample thickness beyond 5 mm can significantly reduce the quality of measurements which do not reflect the bulk stress state.

There are many applications for synchrotron radiation in the analysis of railway components for both residual stress measurements, this technique can also provide phase and microstructural information. The white etching layers on used pearlitic rail was studied by Pyzalla et al. [73]. A 70 mm × 120 mm rail section shown in Figure 8c was used for residual stress analysis at the indicated locations on the worn rail head. For this application, the X-ray penetration depth was required to remain in the white etching layer therefore a depth of 6.1 µm was used. Synchrotron radiation determined the presence of martensite at the rail surface by analysing the change in reflection patterns shown in Figure 8c. A similar study by Wang et al. [74] used Synchrotron techniques for phase analysis complemented by laboratory X-ray diffraction for residual stress measurement. The Synchrotron facilities utilised for analysis of railway components are detailed in Table 1.

#### 3.1.3. Neutron Diffraction

Neutrons have a higher penetration capability, up to 30 mm in steel for internal stress measurements. This allows strain to be non-destructively measured in three principal directions to accurately determine the stress tensor across the sample. For this reason, neutron diffraction finds many applications in the railway industry owing to the ability to acquire triaxial stress with a large sample size capability. Strain scanning is performed by focusing neutrons into a gauge volume at the measurement site, allowing grains that are oriented to meet the scattering condition to diffract the incident neutrons and generate a diffraction peak. This is used to determine the principal strains and stresses. Luzin et al. [79] highlighted three general approaches that utilise neutron diffraction in railway applications. Namely, triaxial 3D stress mapping across full scale rail components, measurement of slices or sections used for comparative analysis and non-destructive surface analysis critical for wear and fatigue investigations that require a greater depth of measurement than can be achieved using other techniques, i.e., X-ray diffraction.

**Figure 9:**
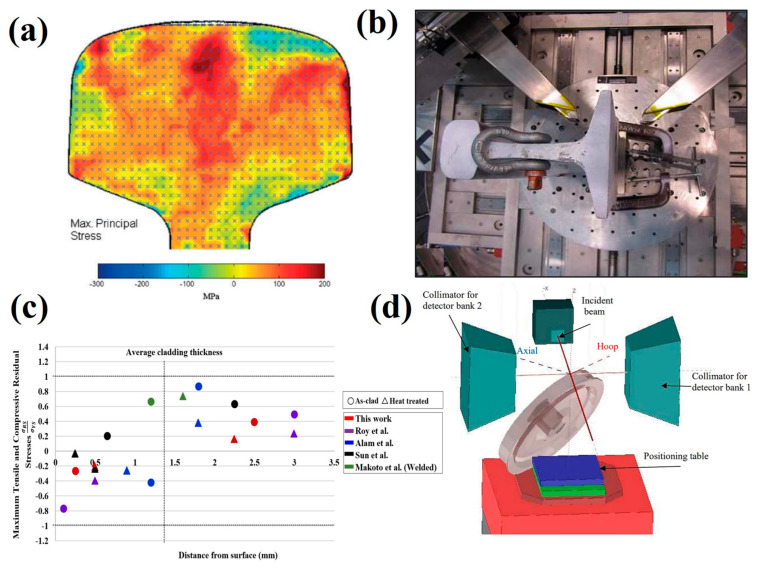
(**a**) Stress map of railhead by Jun et al. [80] (**b**) experimental setup of a rail on a neutron diffractometer stage by Khodabakhshi et al. [83] (**c**) comparison of neutron diffraction obtained measurements in laser clad rail by Kendall et al. with other studies [6] and (**d**) experimental setup for train wheel stress measurements by Alessandroni et al. [86].

Three-dimensional strain mapping was undertaken by Jun et al. [80] on a 16 mm thick cross section taken from a R260 rail. The measurement locations were determined by using a coordinate measurement machine to scan the rail shape before superimposing a grid with 2 mm point spacing to determine the measurement locations. Scans were performed using a 2 × 2 × 2 mm^3^ gauge volume and a resultant stress map is shown in Figure 9a. Non-uniform stresses were identified across the railhead as a result of plastic deformation due to repeated rolling contact loading generating peak compressive sub surface stress that become tensile deeper in the rail head. This was correlated to the full width at half maximum (FWHM) profiles indicating microstructural variations arise because of surface deformation which causes this complex stress state. A similar mapping approach using neutron diffraction was applied by Magiera et al. [81] to determine changes stress during rail manufacture and quantify differences in stress after air cooling, head hardening and roller straightening. Measurements were taken with a 3 × 3 × 3 mm^3^ gauge volume in two directions on 6.5 mm slices assuming plane stress conditions. This 2-dimensional data was then processed to compensate for stress lost during machining to construct 3D stress fields for each stage of rail manufacturing.

Neutron diffraction techniques are particularly effective in the analysis of rail welds and can capture the effects of microstructural changes across the HAZ. Tawfik et al. [82] carried out measurements on a mobile flash-butt welded rail as a means of verifying the internal stresses to validate numerical predictions. A 630 × 170 × 11 mm^3^ plate was extracted from the centreline of a welded AS60 rail. Due to the weld size, a large gauge volume of 3 × 3 × 3 mm^3^ was used for strain measurements at increasing distances from the fusion boundary at three different heights with respect to the rail foot. The stress magnitude was found to increase away from the rail foot, with tensile stresses dominating the rail web after welding. Residual stress in aluminothermic rail welds were assessed to determine the influence of weld stress on fatigue behaviour by Khodabakhshi et al. [83]. The experimental setup for stress measurements across the rail foot is shown in Figure 9b. Strain measurements were taken at 46 locations at the rail foot, across the weld to produce a stress contour plot which established stress increases away from the weld toe and indicated crack initiation sites at the foot of the rail correspond to regions of triaxial and high tensile residual stress.

Extensive strain measurements were undertaken by Roy et al. [84] on hypereutectoid rails after laser cladding with single and double layer deposition of Stellite 6 and 410L. Due the cladding and HAZ thickness of 1.5 mm, a small gauge volume of 0.5 × 0.5 × 10 mm^3^ was used to perform through thickness line scans to ensure this region would fit within the cladding layer. Blind access holes were drilled at the centre of full-scale, laser clad the rails to reduce measurement time and increase neutron signal. Steel laser cladding depositons were found to produce compressive surface stresses whilst tensile residual stresses occurred in Co-Cr based cladding depositions. A post cladding heat treatment was found to significantly reduce peak stresses across the cladding, HAZ and substrate suggesting laser cladding is a promising technology to increase rail fatigue lifetime. Kendall et al. [6] performed neutron diffraction measurements on laser clad rail and compared the effects of heat treatment as shown in Figure 9c. The possibility of using non-destructive methods to obtain microstructural information from analysis of neutron data in laser cladding deposits on high carbon rails was also discussed. The location of the fusion boundary and HAZ were determined by correlating the mircostrain, stress, FWHM and intensity profiles and verified using microstructural analysis. This suggests neutron diffraction can be utilised to obtain strain and microstructural information non-destructively.

Neutron diffraction techniques have also been applied to measure stresses in other complicated railway components. Dhly et al. [85] used neutron diffraction to determine the stress state within axles by strain scanning across a 10 mm thick cross section extracted from a railway axle. Radial measurements were taken by recording the stress profile from the outer surface to the centre, showing compressive stresses at the surface becoming tensile before neutralising at the centre of the axle. Alassandroni et al. [86] undertook neutron diffraction measurements on a 500 kg railway wheel to investigate fatigue cracking. A non-destructive approach was proposed for accurate stress analysis to avoid the stress release which occurs during sectioning techniques; the setup for this is shown in Figure 9d. Line scans were performed along the wheel rim of a complete wheel, using a 4 × 4 × 4 mm^3^ gauge volume. Compressive hoop stresses were determined at the wheel rim surface with compressive or neutral stress in the axial and radial directions, suggesting the manufacturing process to be very effective at producing a desirable stress state. Grosse et al. [87] also applied neutron diffraction techniques for railway wheel analysis. A gauge volume of 2 mm × 10 mm was used to measure strains in full sized railway wheels from 2 mm to 15 mm below the contact surface. The radial strain was found to increase at the surface due to plastic deformation after usage. Rathod et al. [88] applied neutron scattering techniques to 5 mm slices extracted from insulated rail joints (IRJ) to assess severe surface plastic deformation leading to failures. Stresses were measured across a 60 mm^2^ region and analysis of samples exposed cyclic wheel-rail contact highlighted differences between rail deformation at the contact surface produced by in-service loading and the test rig leading to a better understanding of plastic deformation of IRJ and simulation.

Neutron diffraction techniques offer a non-destructive means of obtain internal stress measurements with a high accuracy and resolution. However, two of the main limitations associated with neutron diffraction are the reduced accessibility and costs. This is associated with the highly specialised equipment and technical expertise of instrument scientists required to undertake these measurements. Nonetheless, there are many facilities around the world that provide diffraction instruments to both research and industry and many offer merit-based access to users for beam time. The facilities which have been utilised for stress measurements in the above-mentioned studies are listed in Table 2.

### 3.2. Magnetic

The intrinsic ferromagnetic nature of steels used in the railway industry facilitates the non-destructive measurement of stress using magnetic techniques. Internal strains produce changes in magnetic permeability indicative of residual stress. Under a magnetic field, the ferromagnetic domains within the metal align in the direction of the applied field due to movement of the domain walls. The movement of these domains is influenced by the presence of residual stress therefore the stress state can be determined by measuring parameters such as magnetic permeability, hysteresis and Barkhausen noise.

**Figure 10:**
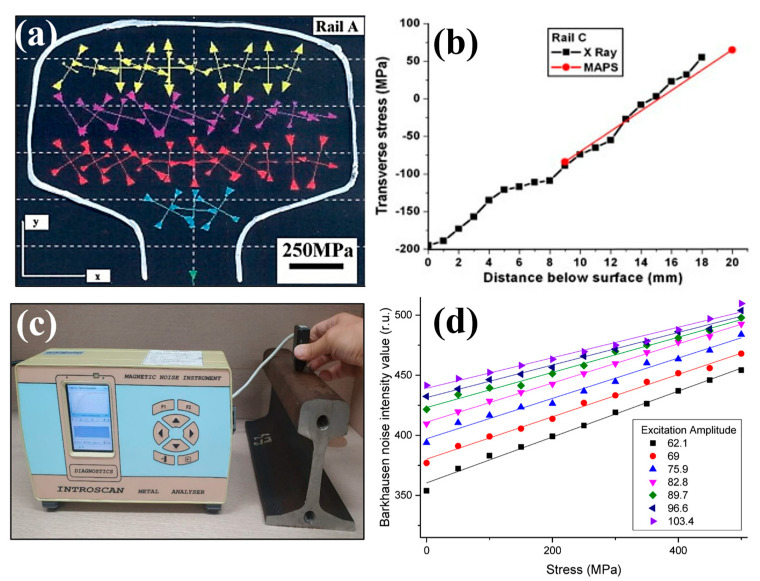
Study by Lo et al. [92] showing (**a**) grid lines of measurements locations and MAPS readings on a railhead, (**b**) comparison of X-ray and MAPS stress measurements. Study by Hwang et al. [93] (**c**) MBN measurements on a rail and (**d**) profile of noise intensity and stress for MBN measurements.

Magnetic anisotropy and permeability system (MAPS) is a portable measurement device which utilises a manual probe to measure magnetic parameters [90]. Buttle et al. [91] performed extensive investigations into MAPS techniques to assess residual stresses in rail in situ. Lo et al. [92] employed this technique to determine residual stresses in new straightened rail and rails with increasing levels of in-service use by performing measurements on transverse and longitudinal cross-sections. The grid showing measurement locations and residual stresses for a transverse cross section is shown in Figure 10a. The new rail contains tensile stresses at the surface whilst in-service loading induces compressive stresses and microstructural hardening in this region. It was observed that due to the size of the probe (10 mm), stresses less than 4 mm below the rail head could not accurately determined without introducing errors by the probe losing contact with the rail. Nonetheless, stress profiles obtained using MAPS show a good agreement with those obtained using X-ray diffraction as indicated in Figure 10b.

The Magnetic Barkhausen Noise (MBN) method measures changes in magnetic domains in response to a changing magnetic field. Electromagnetic pulses are generated due to microstructural discontinuities and strains pining the magnetic domain walls which are detected as the Barkhausen noise. Hwang et al. [93] applied the MBN method to determine stress in passenger rails used in Korea. The experimental setup is shown in Figure 10c where measurements were taken along three regions across the rail head. The authors determined the output signal increased with the applied magnetization. Therefore, 120 Hz was used for stress measurements to produce a sufficient Barkhausen noise output signal. A graph showing the increase in Barkhausen noise intensity with tensile stress is shown in Figure 10d. Neslušan et al. [94] applied this magnetic measurement technique to determine the impact of long term railway wheel operation on surface damage. For this application, residual stresses obtained using surface X-ray diffraction were compared to MBN measurements. Due to increased measuring depth of the magnetic technique, there was not a strong agreement with X-ray obtained stresses. However, changes in grain size due to deformation and elongation had a noticeable effect on the output MBN signal. This therefore recommends the use of MBN techniques for non-destructive analysis of wheel surface condition and has previously been employed in the investigation of white etching layers in rail components [95].

The influence of microalloying additions on rolling contact fatigue in railway wheels was assessed by Rezende et al. [96]. Residual stress measurements using MBN were undertaken at 12 circumferential locations on 39 mm diameter discs extracted from the wheels. The wheels with molybdenum and niobium alloying additions exhibited better wear properties which was attributed to the ability of the microstructure to absorb contact stresses. This was indicated by an increase in MBN noise or tensile stress after testing which was linked to the surface deformation and hardness.

Whilst magnetic techniques for residual stress assessment have proven to be very effective for railway applications, the factors which influence the output signal continues to be of great interest when determining residual stress from MBN. As previously discussed, microstructural changes and deformation due to wear as well as welding and heat treatment influence the Barkhausen noise [97]. Additionally, permeability is known to have a non-linear relationship with stress at high stress magnitudes (i.e., Above 400 MPa), this approach is better suited to components where the internal stress state is known or expected to be low [98]. The MBN effect was implemented by Wang et al. [99] to determine the influence of environmental temperature differences on stress in rails. Higher temperatures were found to decrease measurement features in rail, additionally features which did not vary significantly with temperature were found to have a strong correlation with applied stress. Through this study a relationship was proposed to better understand the influence of temperature and stress distribution on MBN signal. Along with techniques including the contour method, hole drilling and diffraction, Hill Engineering, a USA based company, offers Barkhausen Noise Analysis to measure residual stresses.

### 3.3. Ultrasonic

The ultrasonic residual stress measurement technique is based around the principle that the propagation of ultrasonic waves through a solid material is influenced by the presence of internal stresses. Therefore, by measuring the time of flight of this wave propagation, the internal stresses can be determined non-destructively. There are many advantages associated with this new measurement approach for railway applications including the ability to perform in situ measurements on large components, with rapid measurement times and a measurement depth sufficient to determine a stress gradient. Additionally, compared to other non-destructive diffraction techniques the cost to implement ultrasonic measurements is considerably less due to the availably and transportability of the equipment.

**Figure 11:**
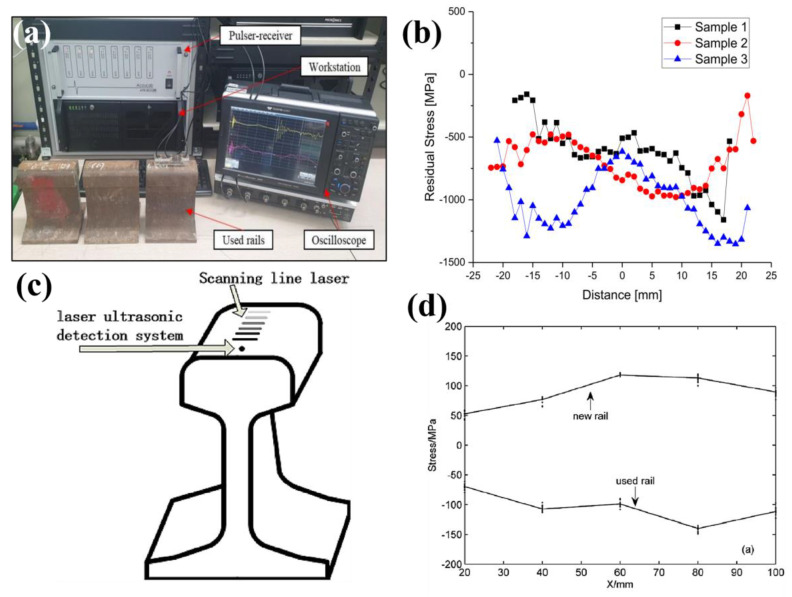
Study by Hwang et al. [37] showing the (**a**) experimental setup for ultrasonic measurements on rails and (**b**) stress profiles for three rail sections. Study by Wang et al. [98] showing (**c**) ultrasonic setup for tread analysis and (**d**) comparison of new and used rail tread stress profiles.

Kudryavtsev et al. [100] investigated residual stresses in a welded railway bridge using portable, ultrasonic residual stress measurement technology. Measurements were taken near the welding site of two 12 mm thick vertical attachments to a bridge section. Tensile stresses up to 240 MPa were determined; after a stress relieving treatment the stresses at the same location were found to decrease to −10 MPa. The ultrasonic technique was applied by Hwang et al. [37] on three sections of KR60 freight rails by taking measurements in 1 mm steps along each rail head using rapidly propagating longitudinal critically refracted waves. The experimental setup for stress measurements is shown in Figure 11a where the acoustoelastic coefficients for this setup had been determined by measuring the wave travel time during tensile testing. The differences between stress in the profiles shown in Figure 11b indicated the rail sections were extracted from different track regions which experienced varying wheel contact which resulted in increased compressive stress in sample 3. Salehi et al. [33] used ultrasonic birefringence to determine residual stress changes in overheated railway wheels through identification of stress relaxation in the wheel rim. The portable measurement system determined of the 65 measured heat-affected wheelsets, over half had retained a desirable compressive stress state making them suitable for operation.

Wang et al. [98] investigated residual stresses in U71 Mn rail tread using a laser ultrasonic approach. The rail head was irradiated using a 10 mm × 0.5 mm source indicated in Figure 11c and collected by a laser ultrasonic detector. The resultant profiles shown in Figure 11d indicate tensile stresses dominate new rail whilst ex-service rails are compressive at the rail tread. It was determined increasing the propagation distance increases the accuracy of the obtained stresses, therefore a distance of at least 20 mm is required in this study. Increasing surface roughness was also found to decrease the stress and produced an error of 41 MPa, indicating surface roughness is an important parameter to consider when implementing ultrasonic measurement techniques. This measurement approach requires specialist technology which can be accessed through groups offering residual stress measurement services. For example, Integrity Testing Laboratory Inc. is a Canadian company offering ultrasonic stress measurement and management capabilities using specialist measuring devices.

To summarize the physical characterisation of the techniques, each method has been compared in Table 3. The advantages and limitations are shown in Table 4 to help the academic and industry users select the appropriate tool to measure residual stresses in rail components.

## 4. Conclusions

This paper presents a review of destructive, semi-destructive and non-destructive stress measurement techniques currently available to assess components, joints and coatings used by the railway industry. The diverse capabilities offered by both destructive and non-destructive approaches comprehensively measures stress in railway track components and railway wheels. These methods have also been implemented to accurately analyse rail axles, bogies, IRJs and crossings as well as high speed train and maglev rail components under a range of welding, coating and joining conditions.

Destructive approaches such as sectioning and hole drilling can be readily implemented on a range of large rail components. Methods such as sectioning and deep hole drilling are regularly complemented by a surface measurement technique to obtain stress measurements ranging from a few microns to tens of millimetres below the surface. This makes destructive techniques particularly suited to rail welds and joins where heat affected regions extend throughout the thickness of the component. Synchrotron and neutron facilities for non-destructive diffraction-based stress measurements are available around the world to users from both research and industry. These facilities have been used to non-destructively assess large and small-scale rail components by enabling surface and internal measurement and the generation of stress maps with resolution high enough to capture the effects of thin rail coatings. Non-destructive approaches such as neutron diffraction, synchrotron radiation and magnetism are further utilised as powerful techniques for microstructural analysis for identification of phases and the locations of microstructural change.

## 5. Future Developments

The future of residual stress measurement for the railway industry lies in the development non-destructive techniques with both high spatial resolution and a broad measurement depth capability. Enhancing the resolution of destructive methods is also of significant interest through use of optical measurement techniques as is developing high resolution EBSD techniques for stress measurement on a granular scale. Each technique has both advantages and challenges and so cross-correlation of the measurements techniques is vital to help address those limitations to provide a better understanding of residual stress in rail components

To achieve a high spatial resolution over a wide measurement depth, residual stress measurement methods for the railway industry are currently used in combination to overcome the limitations of each technique. Significant focus is now aimed towards improving the spatial resolution of individual methods to allow the high resolution currently attainable for surface measurements to be achievable on larger components. This has been done by replacing stain gauges used in hole drilling with optical techniques such as electronic speckle interferometry and digital image correlation, which has the potential for accurate in situ measurements on large industrial components [119,120]. High resolution EBSD is also under development to determine strains across individual grains with a greater spatial resolution than can currently be achieved by non-destructive approaches [121]. Further development of magnetic and ultrasonic methods for in situ stress measurements on full scale infrastructure is of great interest for safe railway operation and improved monitoring practices. Destructive techniques are well documented in standards allowing application in the manufacture of rail components. The continued development of standards for non-destructive residual stress measurement techniques is also critical to allow newer, higher resolution approaches to be utilised more effectively by the railway industry.

Many efforts continue to work towards the accurate simulation of railway manufacturing, welding, coating operations as well as in-service loading which relies on these measured values to determine fatigue behaviour. Therefore, the accurate assessment of residual stresses through experimental measurements will continue to play a vital role in ensuring the safe operation of critical railway infrastructure.

## Figures and Tables

**Table 1:** Synchrotron facilities with rail component residual stress measurement experience.

Facility	Country	Instrument	Experimental Slits Size	Railway Related Studies
APS	United States	34-ID-E	0.3–0.5 µm Horizontal 0.3–0.7 µm Vertical	Dhar et al. [70] Zhang et al. [75]
DESY	Germany	G3 P05	0.1–6000 µm Horizontal 0.1–1200 µm Vertical	Pyzalla et al. [73] Wang et al. [74] Österle et al. [76]
ESRF	France	BM16 and ID11	0.2–1200 µm Horizontal 0.07–1000 µm Vertical	Kelleher et al. [72] Österle et al. [76] Webster et al. [77]
Diamond	United Kingdom	JEEP	Min: 2–13 µm^2^ Max: 50 × 50 µm^2^	Korsunsky et al. [78]

**Table 2:** Neutron facilities with rail component residual stress measurement experience.

Facility	Location	Instrument	Sample Capacity (kg)	Experimental Beam Size	Railway Related Studies
ANSTO	Australia	Kowari	1000	0.2–20 mm horizontal and vertical incident and receiving slits. 2, 3, 5 mm and 10 mm receiving radial collimators.	Kendall et al. [6] Tawfik et al. [82] Khodabakhshi et al. [83] Roy at al. [84] Rathod et al. [88]
FRM-II	Germany	Stress-spec	300	Slits from 0.5 mm up to several mm	Kaiser et al. [56] Jun et al. [80]
ISIS	United Kingdom	Engin-X	1000	0.5–20 mm horizontal and vertical incident and receiving slits. Collimators Sizes: 0.5, 1, 2 and 4 mm	Narayanan et al. [49] Alessandroni et al. [86]
ILL	France	SALSA	500	Slits and collimators arrangements from 0.3 mm up to several mm	Narayanan et al. [49]
NIST	USA	BT-8	100	Collimators Sizes: 0.5, 1, 2 and 4 mm	Luzin et al. [79] Magiera et al. [81]
SINQ	Switzerland	POLDI	200	Slits and collimators Sizes: 0.6, 1.5, 3.8 mm	Grosse et al. [87]
ORNL	USA	HB-2B	50	Slits from 0.3 mm up to several mm	Witt [89]

**Table 3:** Comparison of Physical Characteristics of Residual Stress Measurement Technique.

Technique	Depth	Spatial Resolution	Accuracy	Stresses	Applications	Standards
Sectioning	Not applicable	5 mm	30 MPa steel	Uniaxial/Biaxial	Welds/Joints Large structures Rails, Axles Bogie Frames	EN 13674-1 [101] EN 13262:2020 [102]
Hole Drilling	Approximately equal to the hole diameter (approx. 2 mm)	100 μm	30 MPa steel	Biaxial	Welds/joints Large structures Metal coatings Rails, axles.	ASTM E837-20 [103] NPL Best Practice Guidelines [104]
Contour Method	2–600 mm	300 μm	20 MPa steel	Uniaxial	Medium structures Rails	Best Practice Guidelines by Prime et al. [53] Hosseinzadeh et al. [54]
Laboratory X-ray Diffraction	20 µm Layer removal: 1–4 mm [105]	10 μm	20 MPa steel	Biaxial	Coatings (Laser cladding) Rails, Wheels Axles, Bogies	EN 15305:2008 [106] ASTM E2860 [107]
Synchrotron Diffraction	20 mm in Fe 100 mm in Al	5 μm	10 MPa	Triaxial	Rails Welds Coatings (Laser cladding)	NPL Best Practice Guidelines for X-ray diffraction can also be applied [108]
Neutron Diffraction	30 mm in Fe 100 mm in Al	500 μm	10 MPa	Triaxial	Medium structures Coatings/LMD Welds/joins Rails, wheels, axles, bogies, IRJs	ISO 21,432 [109] Best Practice Guidelines by Daymond et al. [110] IAEA [111]
Magnetic	MAPS 0.1–5 mm [89] MBN 10 μm–1 mm	1 mm	10 MPa	Biaxial	Large structures Rails Wheels Welds/joints	NPL Best Practice Guidelines [112]
Ultrasonic	2–150 mm [113]	5 mm	10 MPa	Biaxial	Large structures (e.g., rail bridges) Wheels Rails Welds/joins	EN 13262:2020 [102]

**Table 4:** Comparison of advantages and disadvantages of residual stress measurement technique.

Technique	Destructive	Cost and Availability	Advantages	Disadvantages	Railway Related Studies
Sectioning	Yes	Low cost, Widely available	Easy to implement Complex shapes and geometry Low resolution	Destructive Low resolution Not for surface measurements	Kang et al. [38] Jun et al. [39] Rieger et al. [40] Schindler et al. [41] Seo et al. [42]
Hole Drilling	Semi	Low cost Widely available	Easy to implement Surface and near surface measurements Incremental measurements	Low resolution Near surface only Wont capture steep stress gradients	Ma et al. [44] Zhu et al. [45] Rao et al. [46] Pokorný et al. [47] Narayanan et al. [49] Ringsberg et al. [50] Moazam et al. [51] Zhu et al. [114]
Contour Method	Yes	Moderate cost Specialised equipment required	No d_0_ required Larger components measured Not effected by microstructure	Destructive Complex post processing Not suitable for high stresses	Kaiser et al. [56] Banerjee [57] Song et al. [115]
Laboratory X-ray Diffraction	No	Moderate cost Specialised equipment required	In situ In-depth measurements with layer removal Accurate surface measurements	Limited to surface Small samples measurement Influenced by surface roughness	Yan et al. [61] Yu et al. [62] Ji et al. [63] Betsofen et al. [64] Rezende et al. [65] Coo et al. [66] Sasaki et al. [67] Regazzi et al. [68] Takahashi et al. [69] Turan et al. [116]
Synchrotron Diffraction	No	High cost Specialised facilities	High Resolution Fast measurement times	Specialised facilities with limited access High level of expertise required	Dhar et al. [70] Dhar et al. [71] Kelleher et al. [72] Pyzalla et al. [73] Wang et al. [74] Zhang et al. [75] Österle et al. [76]
Neutron Diffraction	No	High cost Specialised facilities	High resolution Non destructive Fast data processing Microstructural information	High level of expertise Specialised facilities with limited access Can be semi-destructive	Kendall et al. [6] Narayanan et al. [49] Luzin et al. [79] Jun et al. [80] Magiera et al. [81] Tawfik et al. [82] Khodabakhshi et al. [83] Roy et al. [84] Alessandroni et al. [86] Grosse et al. [87] Rathod et al. [88]
Magnetic	No	Moderate cost Specialised equipment required	In situ Fast measurements Only for ferromagnetic materials	Not suitable for high stresses Influenced by microstructure Surface measurement	Buttle et al. [91] Lo et al. [92] Hwang et al. [93] Neslušan et al. [94] Rezende et al. [96] Balanovsky et al. [97] Wang et al. [99]
Ultrasonic	No	Moderate cost Specialised equipment required	Portable Surface or through thickness measurements	Calibration coefficients required	Hwang et al. [37] Wang et al. [98] Kudryavtsev et al. [100] Salehi et al. [33] Hwang et al. [117] Murav’ev et al. [118]

## Data Availability

Not applicable.
